# Radiosurgical Treatment Verification Using Removable Megavoltage Radiation Detectors

**DOI:** 10.7759/cureus.1889

**Published:** 2017-11-29

**Authors:** Cesare H Jenkins, Robert Kahn, Georg A. Weidlich, John R. Adler

**Affiliations:** 1 Engineering, Zap Surgical Systems; 2 Radiation Oncology, National Medical Physics and Dosimetry Comp., Inc; 3 Department of Neurosurgery, Stanford University School of Medicine

**Keywords:** portal dosimetry, treatment verification

## Abstract

Introduction

Accurate dose delivery is critical to the success of stereotactic radiosurgery. Unfortunately, verification of the accuracy of treatment delivery remains a challenging problem. Existing radiosurgery delivery paradigms are limited in their ability to verify the accurate delivery of radiation beams using data sampled from the beam after it has traversed the patient. The Zap-X Radiosurgery System (Zap Surgical Systems, San Carlos, CA) addresses this issue by implementing a fully integrated treatment delivery system that utilizes a factory commissioned megavoltage (MV) imager to measure the transmitted beam. The measured intensity is then compared with an expected value in order to confirm that treatment is proceeding as expected. The purpose of this study was to evaluate a prototype system and investigate the accuracy of an attenuation model used in generating the expected transmitted intensity values.

Methods

A prototype MV imager was used to measure transmitted beam intensities at various exposure levels and through several thicknesses of solid water. The data were used to evaluate imager linearity and model accuracy.

Results

Experimental results indicate that a quadratic attenuation model is appropriate for predicting beam attenuation and that the imager exhibits excellent dose linearity.

Conclusions

The MV imager system is shown to be capable of accurately acquiring the data needed to confirm treatment validity.

## Introduction

Radiosurgery is a critical tool for treating a broad set of benign and malignant lesions, particularly in the head and neck region of the body [1-3]. Fundamental to the clinical efficacy of radiosurgery is the accurate delivery of high doses of ionizing radiation. While there are means for detecting how much radiation is emitted from a source, for example, a linear accelerator (LINAC), current treatment modalities are open-loop systems providing little or no feedback as to the accuracy of radiation actually administered to the patient. In the event of system miscalibration, this limitation permits serious treatment errors to go undetected until well after treatment has concluded [4-5].

The first line of defense against erroneous radiation delivery is a rigorous quality assurance program that addresses all aspects of the treatment workflow [6-7]. While such measures are appropriate, valuable, and important, there remain areas wherein additional improvement can be made in providing verification of accurate treatment delivery.

The general treatment paradigm for all stereotactic radiosurgery systems is to deliver a predetermined amount of radiation from several different locations surrounding the patient’s head. The amount of radiation delivered from each of these locations is controlled using a monitor chamber (LINAC-based systems) or timer (Cobalt-based systems). In either case, an initial calibration is required to determine the appropriate relationship between the onboard measurement device and deposited dose. Performing this calibration is a complicated procedure that is sensitive to user error [8]. In some instances, this calibration must be checked or repeated periodically. The complexity (and at times, the repetitious performance) of these procedures make them vulnerable to user error [4].

In addition, the measurement of dose output occurs before the beam passes through collimators that ultimately shape the beams to match the tumor anatomy. This means that errors in the size or positioning of these collimators will not be captured in the machine’s dose output measurement. 

Finally, while radiosurgery systems universally employ a patient positioning system, these are typically operated as independent systems, with no feedback from the beam used to verify proper patient positioning. Again, this creates the possibility of having calibration errors (calibration of the patient positioning system relative to the treatment delivery system) remaining undetected throughout treatment. In addition, these systems are generally not designed to detect changes in patient anatomy that occur between planning and delivery. While some of these changes will not have a clinical impact, others could affect the accuracy of the delivered dose.

Once a treatment beam has traversed the patient, the residual dose is a function of the initial delivered dose, collimation, and both the amount and specific nature of patient tissue traversed. Thus, measuring and analyzing the beam after it has exited the patient provides a unique opportunity to verify the accuracy of all aspects of treatment delivery. While spatially sensitive transmission chambers offer feedback on the accuracy of output and collimation, only traditional LINAC-based radiosurgery platforms equipped with an electronic portal imaging device (EPID) offer the opportunity to capture and analyze information about the beam after it has traversed the patient [9-10]. Such systems still require extensive user commissioning, are often limited to offline analysis, and have seen limited clinical acceptance to date.

The Zap-X Radiosurgery System (Zap Surgical Systems, San Carlos, CA) is a novel first-of-a-kind self-shielded device designed specifically for brain as well as head and neck radiosurgery. To address the above issues, the Zap-X Radiosurgery System (Zap-X) seeks to implement a method of using a factory-calibrated megavoltage (MV) detector to examine the exit dose of each delivered beam in real time, and thereby ensure treatment is proceeding as expected. In this paper, we discuss the theory of operation for such a system and present proof of concept data.

The Zap-X Radiosurgery System and MV imager seek to validate the accuracy of delivery by performing a real-time comparison of measured and expected transmitted beam intensity. To accomplish this task, there are two required components: (1) a measurement of the transmitted intensity for each individual treatment and (2) a prediction of the expected transmitted intensity. The Zap-X System utilizes a removable MV imager designed to be replaced at regular intervals. Each imager is fully calibrated and commissioned at the factory. The detector provides a real-time view of transmitted radiation that is depicted on the user interface during radiosurgery as well as a measurement of the transmitted intensity at the center of the treatment beam.

As a source for comparison, the Zap-X treatment planning system calculates the expected transmitted dose for each beam in a treatment plan. While the transmitted beam intensity is clearly a function of output and the attenuation, the exact model for predicting attenuation is non-trivial. The basic physics model of x-ray attenuation is commonly expressed as 

$$I=I_0e^{-\mu x}$$

where $I$ is the transmitted intensity, $I_0$ is the original intensity, $\mu$ is the attenuation coefficient of the material being transited, and $x$ is the thickness of material through which the beam has passed. Unfortunately, this model assumes that all photons in the beam have the same energy and that the attenuating material is entirely uniform. Since neither of these assumptions holds in the case of radiosurgery, it would be necessary to calculate the sum over all materials and all photon energies within the beam. Since this becomes rather impractical, several empirical models have been derived to approximate the resulting transmission.

Swindell proposed the following formula as a model for polychromatic x-ray transmission through a water-equivalent volume [11] 

$$I=I_0e^{-Ax-Bx^2}$$

where $A$ is analogous to the linear attenuation coefficient, $\mu$ , from the equation above, and $B$ is an empirically derived quadratic factor that captures the behavior of the multispectral photon interactions within the patient.

The purpose of this paper is to evaluate the accuracy of such a model in the context of the Zap-X Radiosurgery System. 

## Materials and methods

**Figure 1:**
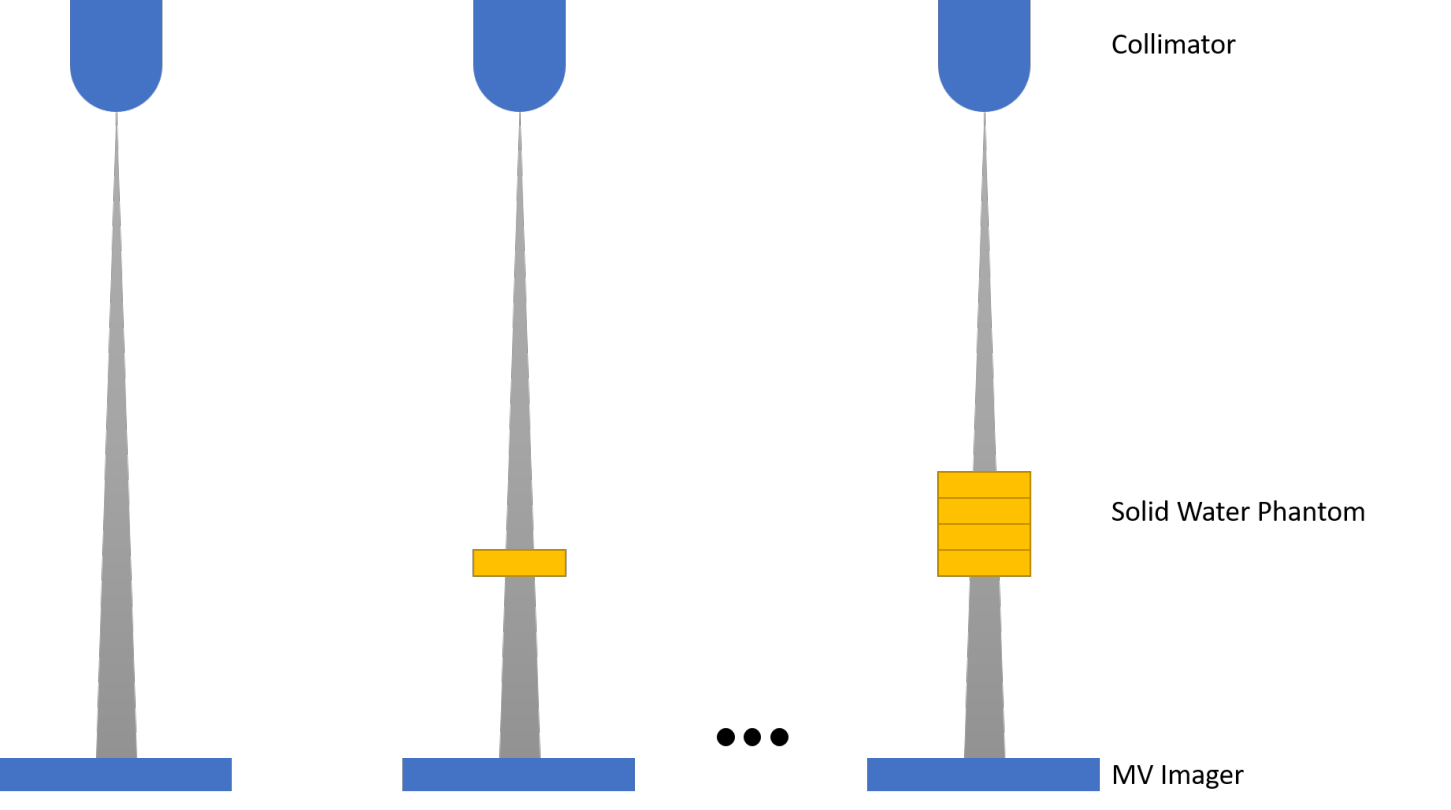
Experimental setup for measuring transmitted dose through various thicknesses of a solid water phantom.

To determine the parameters of the attenuation model, 10 beams of 100 MU were delivered with nothing in the beam path or through a solid water phantom of various thicknesses (5, 10, 16, and 22 cm) using a Zap-X Radiosurgery System equipped with a prototype MV imager (see Figure 1). The measurements were then normalized to either the empty or 10 cm attenuation point. The parameters were then found by minimizing the sum of squared differences between the model and the measured data using a GRG non-linear optimization algorithm.

The resulting model was then evaluated by delivering 10 beams of 10, 20, 50, 100 and 200 MU at each of the attenuation thicknesses noted above. This range of MU per beam represents typical values for the Zap-X Radiosurgery System.

To evaluate the model, it is necessary to transform measurements from the MV imager into units consistent with other elements in the system. For the purposes of this study, the decision was made to evaluate the model by transforming all measurements into units of MU. The comparison to be made is between the MU measured by the onboard monitor chamber, which utilizes the system calibration, and a predicted MU value, derived by way of a calibration factor and attenuation model, from the MV image intensity values.

**Figure 2:**
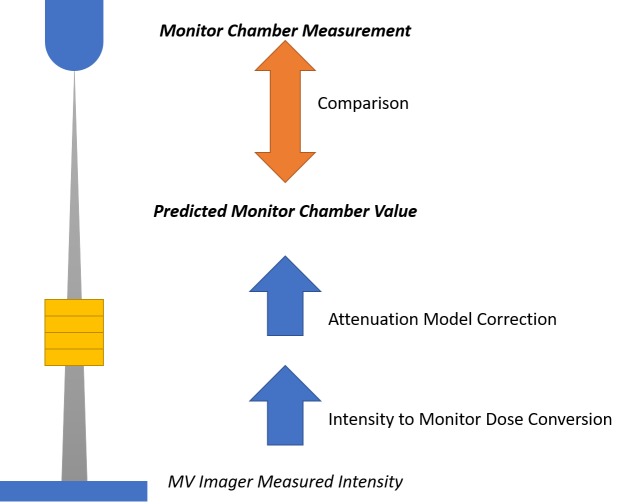
Schematic of data collection and comparison for model validation. Measurements from the MV imager are converted to monitor chamber dose using a calibration factor and corrected for anticipated attenuation using the determined model. The resulting predicted monitor chamber value is then compared with the monitor chamber measurement for each beam.

A linear fit was performed to determine a calibration factor relating measured intensities to the dose reported by the onboard monitor chamber. Then, for each beam, the measured intensity, calibration factor, and attenuation model were used to estimate the monitor chamber reading. The estimated and actual chamber readings were then compared (see Figure 2). Subsequently, the average and standard deviation of the resulting errors were calculated. 

## Results

**Figure 3:**
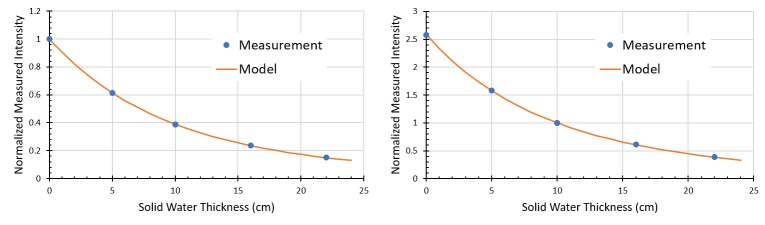
Comparison of attenuation models developed with different normalization conditions. Attenuation models developed for measurements normalized to no attenuation (left) and 10 cm of solid water (right). Each measurement point represents the mean of ten measurements with 100 MU exposure.

**Figure 4:**
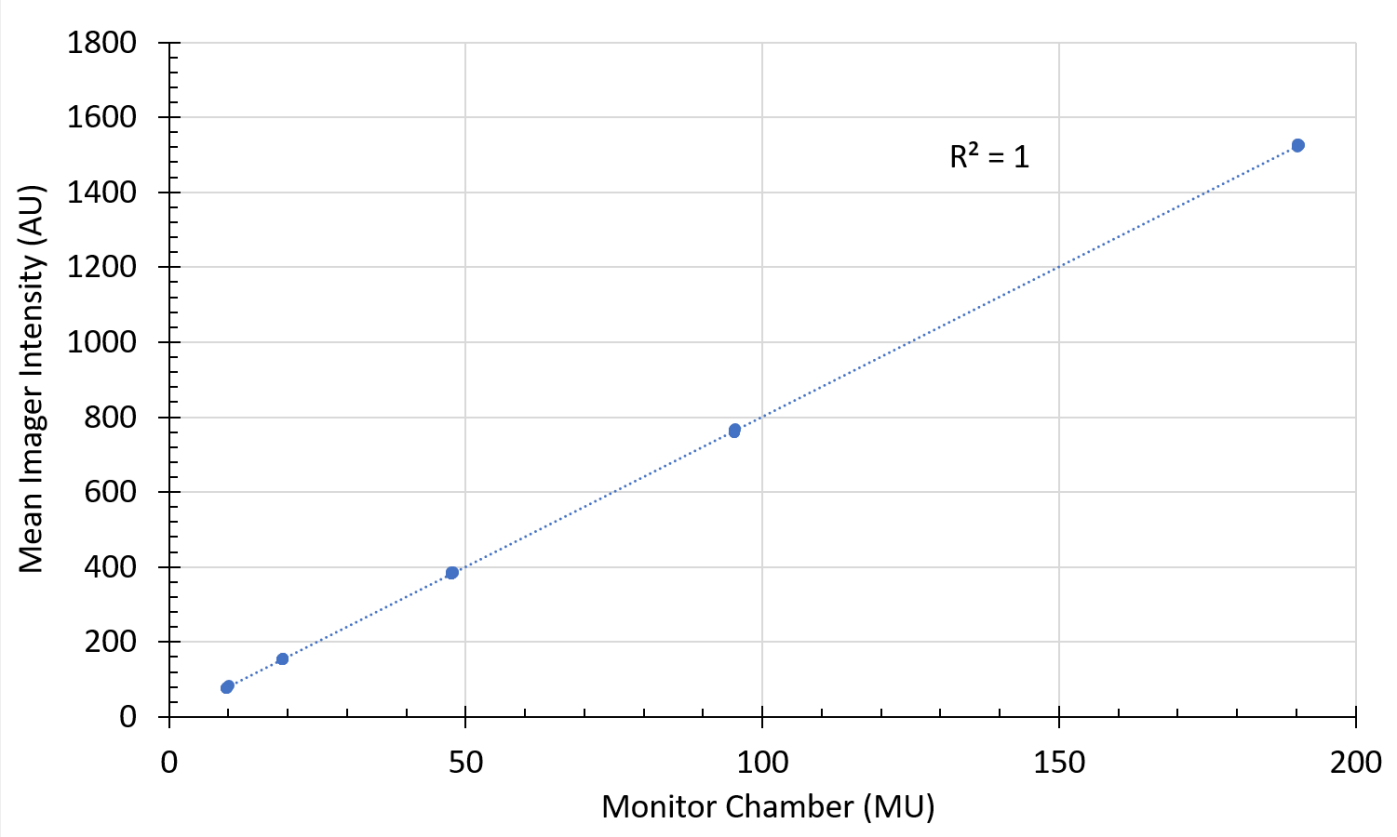
Imager dose linearity. Displayed data were acquired through 10 cm of solid water.

Normalized intensity values, after transiting various thicknesses of water equivalent plastic, are shown in Figure 3. The sum of squared residuals was $6.9\times 10^{-5}$ and $5.7\times 10^{-6}$ for the zero attenuation and 10 cm attenuation models respectively. Table 1 and Table 2 present tabular results for the model evaluation. The average predictive error across all conditions was 0.65% and 0.01% for the 0 cm and 10 cm models. Figure 4 offers a brief view of dose linearity of the system. This linearity experiment was repeated after delivering several thousand MU and no significant differences were observed.

**Table 1:** Predictive errors for the attenuation model created with values normalized to zero attenuation.

	Predictive errors for attenuation (cm)				
MU/Beam	0	5	10	16	22
10	0.1% (0.67%)	0.4% (1.17%)	-1.6% (1.42%)	-2.2% (1.93%)	-4.4% (2.44%)
20	-0.2% (0.26%)	-0.4% (0.49%)	-0.9% (0.66%)	-1.0% (1.63%)	-4.5% (1.23%)
50	0.1% (0.15%)	0.1% (0.35%)	-0.3% (0.38%)	0.0% (0.57%)	-1.0% (0.84%)
100	0.0% (0.13%)	0.2% (0.19%)	-0.3% (0.35%)	0.3% (0.29%)	-0.6% (0.57%)
200	0.0% (0.13%)	0.1% (0.18%)	-0.1% (0.18%)	0.7% (0.49%)	-0.8% (0.64%)

**Table 2:** Predictive errors for the attenuation model created with values normalized to 10 cm of attenuation.

	Predictive errors for attenuation (cm)				
MU/Beam	0	5	10	16	22
10	0.4% (0.68%)	1.4% (1.19%)	0.1% (1.45%)	0.6% (1.98%)	-0.3% (2.52%)
20	-0.1% (0.27%)	0.0% (0.51%)	0.0% (0.69%)	0.4% (1.70%)	-2.6% (1.28%)
50	-0.1% (0.15%)	0.3% (0.37%)	0.0% (0.40%)	0.6% (0.60%)	-0.4% (0.88%)
100	-0.2% (0.14%)	0.3% (0.20%)	-0.1% (0.37%)	0.6% (0.31%)	-0.4% (0.60%)
200	-0.3% (0.14%)	0.1% (0.19%)	0.0% (0.19%)	0.9% (0.51%)	-0.8% (0.67%)

## Discussion

The Zap-X system seeks to implement an integrated and streamlined system for treatment verification using a factory calibrated MV imager. By providing an independently calibrated check of the administered dose, such a system affords an extra layer of safety. Being a transit dosimetry system, the Zap-X MV imager gives the user real-time feedback on the overall accuracy of the complete treatment, including the accuracy of output as well as collimation in a streamlined integrated fashion, without requiring the user to calibrate the detector. In the future, this technology may be useful in identifying gross deviations in patient anatomy (e.g. swelling, weight loss, and patient movement on the table), in a streamlined integrated fashion, without requiring the user to calibrate the detector.

For such a system to be successful, an accurate imager calibration and model of beam attenuation are needed. A primary concern in developing these calibrations and models is the conditions under which the models are derived. Figure 3 shows that deriving the model based on zero attenuation and 10 cm of water equivalent attenuation yield similar results. Tables 1-2 indicate a similar result. While some differences are observed, particularly at higher levels of attenuation, and smaller numbers of MU/beam, these differences are not statistically significant (p<0.05). Overall, the fact that average predictive errors were not significantly different from zero indicates that the proposed model is sufficient for predicting the attenuation of the beam.

The slight errors at the normalization point in Tables 1-2 are due to errors in the imager intensity to monitor chamber dose calibration, as this was performed with a linear fit without a specific normalization point.

In addition to attenuation model accuracy, Figure 4 indicates that the imager exhibits excellent dose linearity. This behavior was confirmed before and after large amounts of radiation exposure.

Moving forward, additional characterization of the detector will take place. Specifically, detector repeatability will need to be evaluated, as well as the consistency of the beam attenuation model, with different collimator sizes, over time, and across machines.

## Conclusions

A quadratic exponential decay model provides an accurate estimate of beam attenuation for an integrated transit dosimetry technique on the Zap-X radiosurgery system. In addition to the accuracy of the attenuation model, the system is also shown to be linear with dose. The accuracy of such a model is required for the system to be capable of detecting errors in treatment delivery, including errors in machine output calibration, beam collimation and, in the future, differences in patient anatomy compared with what was anticipated by the treatment planning system.
